# Characterization of Intracellular and Extracellular Saxitoxin Levels in Both Field and Cultured *Alexandrium* spp. Samples from Sequim Bay, Washington

**DOI:** 10.3390/md20080006

**Published:** 2008-05-14

**Authors:** Kathi A. Lefebvre, Brian D. Bill, Aleta Erickson, Keri A. Baugh, Lohna O’Rourke, Pedro R. Costa, Shelly Nance, Vera L. Trainer

**Affiliations:** 1 NOAA Fisheries, Northwest Fisheries Science Center, Marine Biotoxins Program, 2725 Montlake Blvd. East, Seattle, WA, 98112, USA; E-mails: Kathi.Lefebvre@noaa.gov; Brian.D.Bill@noaa.gov; Keri.Baugh@noaa.gov; Shelly.Nance@noaa.gov; Vera.L.Trainer@noaa.gov; 2 Jamestown S’Klallam Tribe, 1033 Old Blyn Highway, Sequim, WA, 98382, USA; E-mails: aerickson@jamestowntribe.org; lorourke@jamestowntribe.org; 3 IPIMAR, National Institute for Agronomy and Fisheries Research, Av. Brasília, 1449-006 Lisbon, Portugal; E-mail: Pedro.Costa@noaa.gov

**Keywords:** PSP, PSTs, harmful algal blooms, saxitoxin, *Alexandrium* spp., extracellular toxins, intracellular toxins, HAB

## Abstract

Traditionally, harmful algal bloom studies have primarily focused on quantifying toxin levels contained within the phytoplankton cells of interest. In the case of paralytic shellfish poisoning toxins (PSTs), intracellular toxin levels and the effects of dietary consumption of toxic cells by planktivores have been well documented. However, little information is available regarding the levels of extracellular PSTs that may leak or be released into seawater from toxic cells during blooms. In order to fully evaluate the risks of harmful algal bloom toxins in the marine food web, it is necessary to understand all potential routes of exposure. In the present study, extracellular and intracellular PST levels were measured in field seawater samples (collected weekly from June to October 2004–2007) and in *Alexandrium* spp. culture samples isolated from Sequim Bay, Washington. Measurable levels of intra- and extra-cellular toxins were detected in both field and culture samples via receptor binding assay (RBA) and an enzyme-linked immunosorbent assay (ELISA). Characterization of the PST toxin profile in the Sequim Bay isolates by pre-column oxidation and HPLC-fluorescence detection revealed that gonyautoxin 1 and 4 made up 65 ± 9.7 % of the total PSTs present. Collectively, these data confirm that extracellular PSTs are present during blooms of *Alexandrium* spp. in the Sequim Bay region.

## 1. Introduction

**Figure 1:**
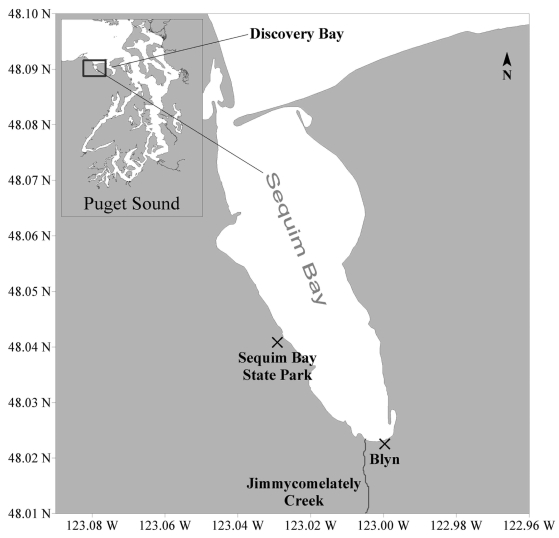
Map shows the locations of seawater and shellfish collection sites in Sequim Bay, Washington. Seawater samples were collected from the pier at Sequim Bay State Park. Shellfish sampling sites for the Washington State Department of Health biotoxin monitoring program include Sequim Bay State Park and Blyn. Discovery Bay is the first embayment to the east of Sequim Bay in the map inset.

The deaths of three people and illness of two others in 1942 after their ingestion of mussels and butter clams collected from Sekiu beach, about 90 mi west of Sequim Bay, provided the first evidence for the presence of high levels of paralytic shellfish toxins (PSTs) in Washington State ^[1]^. Monitoring for PSTs in Washington became formalized in 1957 after another large outbreak of paralytic shellfish poisoning (PSP) occurred in neighboring British Columbia, Canada ^[2]^. The suite of PSTs consists of saxitoxin (STX) and its derivatives, including neosaxitoxin (NEO), decarbamoyl STX (dcSTX), gonyautoxin 1 and 4 (GTX1,4), GTX2,3, dcGTX2,3, dcNEO, GTX5, and the sulfamate saxitoxins. Saxitoxin is the most potent of the PSTs and for monitoring purposes, PSTs are quantified in terms of STX equivalents. During 1957, the first shellfish closure occurred in Sequim Bay when PST levels as high as 162 μg STX equiv./100g shellfish were measured in butter clams. The maximum decadal abundances of PSTs during the fifties and sixties were restricted to the northern margins of Puget Sound (e.g., in Sequim and Discovery Bays; Figure 1) but then expanded to the San Juan Islands and central Puget Sound waters by the seventies and eighties ^[3]^. Sequim Bay therefore has the longest documented record of shellfish toxins in Washington State. Shellfish closures have occurred annually in Sequim Bay which is considered to be a Puget Sound “hot spot” site, one of the three monitored locations in northern Puget Sound that most frequently have the highest levels of PSTs in mussels ^[4, 5]^.

Sequim Bay and its major tributary, Jimmycomelately Creek, are home to a run of summer chum salmon (now nearly extinct) as well as coho salmon, winter steelhead, and cutthroat trout ^[6]^. Sequim Bay is also a major herring spawning ground and the home of other marine fauna such as the sand lance, native littleneck clams, bald eagles, osprey, kingfishers, shorebirds and otters. Many citizens of the Jamestown S’Klallam tribe are dependent upon the natural resources of the Bay for a portion of their livelihood. Finfish, clams, geoduck, crab and shrimp are harvested for the commercial market, and all are culturally important resources harvested for subsistence and ceremonial purposes. The long-term record of PSTs in Sequim Bay and the economic and cultural importance of fisheries to the people who live on its shores make it an ideal site to measure *in situ* concentrations of extra- and intracellular PSTs in order to characterize their interannual variability and assess potential exposure risks to the marine food web.

Recent work has shown that exposure to STX that was directly dissolved in freshwater and seawater impairs sensorimotor function in the larvae of ecologically and commercially important fish species ^[7, 8]^. However, in the past, concentrations of extracellular PSTs have not been measured in the field due to both the lack of sensitive methods and the unpredictability of harmful algal blooms. The focus to date has been on intracellular toxin concentrations *in situ*; therefore ecologically relevant concentrations of extracellular toxins are poorly characterized. In laboratory cultures, extracellular PST concentrations have been measured from the culture media of cyanobacterial isolates (*Anabaena circinalis*) at levels of ~75 μg/L, providing evidence that extracellular toxins are present for at least some species ^[9]^. Fortunately, a highly sensitive enzyme-linked immunosorbent assay (ELISA) for PST detection has recently become available thru Abraxis LLC (Warminster, PA), enabling us to pursue characterization of toxin levels in marine waters. In the present study, we quantified PST levels (both extracellular and intracellular) and *Alexandrium* spp. cell densities in water from Sequim Bay during summers for four years (2004–2007). Additionally, we compared our PST and cell density data to shellfish PST data obtained by the Washington State Department of Health over the same time period. We also performed laboratory culture studies with Sequim Bay *Alexandrium* spp. isolates in order to validate toxin detection methods, verify toxin production levels, and identify PST profiles in local species.

## 2. Materials and Methods

### 2.1 Collection of Sequim Bay seawater samples

Seawater samples used for quantifying intracellular and extracellular STX levels, and *Alexandrium* spp. cell densities were taken on a weekly basis from the Sequim Bay State Park dock from June to October 2004 – 2007 (Figure 1). Seawater was obtained from a depth of 1 meter using a Niskin bottle. For intracellular STX samples, 1.5 L were vacuum filtered on 47 mm diameter (0.45 μm pore size) HA membrane filters (Durapore Millipore Inc.). The filters were folded and frozen in 15 ml Falcon tubes (BD Biosciences). To collect samples for extracellular STX analyses, 1 ml of 0.45 μm filtrate was collected in microfuge tubes and frozen at −20°C. HA filters are made of mixed cellulose esters and do not bind STX, making them useful for filtering soluble STX ^[10]^. Raw seawater samples were preserved in buffered formalin (1–2%) in 125 ml glass jars and stored for quantification of *Alexandrium* spp. cell densities.

### 2.2 Toxin and cell quantification

Frozen filters were extracted with 5 ml of dH_2_O for intracellular STX quantification. To ensure complete extraction, filters were ground by hand with a spatula (Fisherbrand Chemi-Scraper, www.fishersci.com) and vortexed for 30 s. Samples were then sonicated in a Branson 5510 ultrasonic cleaner for two one-hour intervals and vortexed for 30 s after sonication steps. Intracellular STX levels were quantified using a receptor binding assay (RBA) as described in Trainer and Poli (2000). Extracellular STX samples collected in 2004 were also analyzed by RBA ^[11]^, but required a minimum dilution of 1:40 in binding buffer to eliminate matrix effects. After 2004, extracellular STX samples were analyzed via ELISA (Abraxis LLC, Warminster, PA). For extracellular STX quantification via ELISA, frozen filtrate was thawed and analyzed at 1:25 in the dilution buffer provided with the kit. Cell densities of *Alexandrium* spp. were determined via manual cell counts. Prior to counting, the cells in the preserved seawater samples were allowed to settle overnight. The overlying seawater was then aspirated and the remaining volume containing the settled cells was measured. 100 μl of the settled sample was placed in a Palmer-Maloney nanoplankton slide and counted by eye using a microscope. The number of cells counted per 100 ul was converted to cells per L after dividing by the settling concentration factor (usually 10X).

### 2.3 Isolation and analysis of laboratory culture samples for growth studies

To establish uni-algal laboratory cultures, single *Alexandrium* spp. cells or chains were isolated from phytoplankton net tow (20 μm mesh size) collections and maintained in f/2 (as described in ^[12]^) nutrient enriched seawater batch cultures at 12°C, on a 12 hr light: 12 hr dark cycle at approximately 80–120 μE/m^2^ s. Cell densities, intracellular STX, and extracellular STX were quantified throughout the growth cycle ( 30 to 46 days) in three cultures (collected in summer 2004) following the same methods as those described for field-collected samples, with the exception that only 20 ml were filtered for intracellular STX and no settling was required for manual cell counts.

### 2.4 Quality control experiments for extraction and filtering techniques

A comparison of extraction efficiencies between various extraction protocols was performed using eight sets of duplicate intracellular STX culture samples that were extracted with dH2O and 0.1 M acetic acid. Cells from eight different cultures were filtered twice (50 ml each) as described above for intracellular STX to generate 16 intracellular STX filters (8 for dH_2_O and a duplicate set of 8 for 0.1 M acetic acid extraction). The filters were frozen in 15 ml Falcon centrifuge tubes until extraction and analysis via receptor binding assay. Each extract was further split and extracted with boiling HCl as described in Ravn *et al.*, 1995 ^[13]^. Multiple paired *t*-tests (significance level of P < 0.05) were used to determine if there were statistically significant differences between intracellular STX values quantified in samples extracted via dH_2_0 and intracellular STX values from samples extracted via acid treatments.

In order to verify that extracellular STX values quantified in dense culture samples (> 1 x 10^6^ cells/L) were not influenced by cell lysis during vacuum filtration, a quality control experiment was performed with five dense culture samples. Duplicate sets of five cultures were filtered (50 ml) under normal (5 in. Hg) and light (2 in. Hg) vacuum pressure. The filtrate was analyzed for extracellular STX using receptor binding assay and the presence of chlorophyll was used as an additional signal for cell lysis. Chlorophyll was measured in relative fluorescence units (RFU) on a Turner Designs 10-AU fluorometer. A *t*-test (significance level of P < 0.05) was used to determine if there were significant differences in extracellular STX values between normal and light vacuum treatments.

### 2.5 Characterization of toxin profiles in Sequim Bay Alexandrium spp. isolates

A set of four *Alexandrium* spp. isolates from Sequim Bay, Washington were grown to densities of >6 X 10^6^ cells/L in laboratory cultures and analyzed for the suite of PSTs by the AOAC approved HPLC precolumn oxidation method for the detection of PSP toxins in shellfish ^[14]^. Cell densities were quantified and intracellular toxin samples were extracted as described above for field-collected samples, with the exception that only 50 ml were filtered and no settling was required for manual cell counts. In addition to intracellular STX quantification by HPLC, both extracellular and intracellular STX samples from these four isolates were also quantified via receptor binding assay (RBA) and ELISA in order to compare toxin detection methods. A set of standards (Neosaxitoxin (NEO), decarbamoyl saxitoxin (dcSTX), gonyautoxin 1 and 4 (GTX1,4), GTX2,3, dcGTX2,3, dcNEO, B1, and C1,2; National Research Council, Halifax, NS, Canada) were also analyzed by RBA to determine the % reactivity (as compared to STX) for each toxin type. Three of the toxin standards (GTX1,4, GTX2,3, and dcGTX2,3) were analyzed at three concentrations to determine the standard deviation of the assay.

## 3. Results

### 3.1 Alexandrium spp. cell densities and toxin presence in field-collected seawater

**Figure 2:**
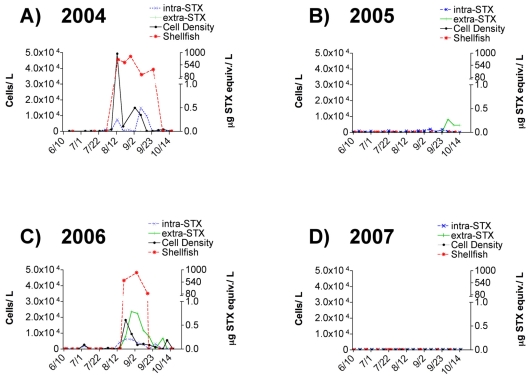
*Alexandrium* spp. cell densities, intracellular saxitoxin levels, and extracellular saxitoxin levels measured in surface waters collected from the pier at Sequim Bay State Park over four consecutive years from June – October. The graph also shows paralytic shellfish toxin (PST) levels quantified by the standard mouse bioassay in shellfish species (mussels, manila clams, and oysters) collected from Sequim Bay State Park and Blyn (shellfish sample sites are shown in Figure 1) by the Washington State Department of Health for the same time period. The left y-axis depicts cell density (cells/L) and the right y-axis depicts toxin concentrations (μg STX equiv./L for intracellular STX and extracellular STX, and μg STX equiv./100 g for shellfish). The regulatory limit for PSTs in shellfish is 80 μg STX equiv./100 g and is indicated at the break in the right y-axis.

*Alexandrium* spp. blooms were observed in Sequim Bay during two alternating years of the four-year study period. Maximum cell densities during the sampling period (June–October) reached 5 x 10^4^ and 2 x 10^4^ cells/L in years 2004 and 2006, respectively, but were undetectable in years 2005 and 2007 (Figure 2).

*Alexandrium* spp. blooms occurred every other year, but consistently during the months of August and September (Figure 2), which is historically typical for Sequim Bay, Washington based on shellfish toxicity records. PST values measured in shellfish by the Washington State Department of Health (WDOH) from 2004 to 2007 correlated with the presence of *Alexandrium* spp. and intracellular STX values throughout the entire study (Figure 2). PST values in shellfish surpassed regulatory limits (80 μg STX equiv./100g shellfish) during both bloom periods (Figure 2A, C). Maximum intracellular STX values reached 0.5 and 0.2 μg STX equiv./L in 2004 and 2006 respectively. In addition to intracellular STX, detectable levels of extracellular STX, as measured by ELISA, were observed in 2006. In fact, a maximum extracellular STX value of approximately 0.8 μg STX equiv./L was observed in early September 2006, just after the peak *Alexandrium* spp. cell density and was higher than corresponding intracellular STX values (Figure 2C). Unfortunately, in 2004 the ELISA kit was not available and intracellular STX samples were only analyzed by receptor binding assay (RBA). The RBA was not sensitive enough to detect extracellular STX in 2004 because the samples could not be analyzed at dilutions below 1:40 due to matrix effects. Although cells were not observed in manual cell counts during 2005, low intracellular STX values ranging from 0.02 to 0.06 μg STX equiv./L were detected by RBA and extracellular STX levels ranging from 0.15 to 0.28 μg STX equiv./L were quantifiable by ELISA during late September (Figure 2B).

### 3.2 Alexandrium spp., cell densities and toxin presence in culture growth studies

**Figure 3:**
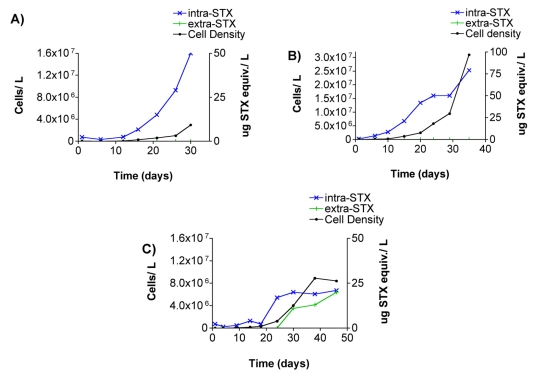
*Alexandrium* spp. cell densities and intracellular and extracellular saxitoxin levels measured in three uni-algal batch cultures. The left y-axis depicts cell density (cells/L) and the right y-axis shows toxin concentration (μg STX equiv./L). Detectable levels of extracellular STX as measured by receptor binding assay (RBA) were only observed in culture flask C. Cell density and toxin scales vary for clarity.

Intracellular STX and high densities of *Alexandrium* spp. were present in all three grow out experiments with Sequim Bay culture isolates (Figure 3). Incubation periods were 30, 35, and 46 days for cultures A, B, and C, respectively. Cell densities and intracellular STX were correlated in all three cultures. Extracellular STX was only detected in culture C (Figure 3C). ELISA kits were not available at the time of the culture grow out experiments, and consequently extracellular STX was measured by receptor binding assay. With the use of an f/2 culture media blank, extracellular STX values in culture C were high enough to distinguish from matrix effects at dilutions of 1:40.

### 3.3 Results of quality control experiments for extraction and filtration procedures

There were no significant differences in intracellular STX values quantified in samples extracted via dH_2_O and samples extracted via acid treatments (Table 1). Consequently, all other field-collected and cultured samples used for intracellular STX quantification in the study were extracted with dH_2_0. Additionally, there were no significant differences in extracellular STX values in duplicate samples filtered via normal and light vacuum pressure (Table 2). Chlorophyll was not detected in either set of vacuum-filtered extracellular STX samples, indicating that cell lysis had not occurred. These data suggest that our vacuum filtration procedures did not cause cell lysis and that our reported extracellular STX values reliably represent natural field and culture media levels.

### 3.4 Toxin profiles in Sequim Bay Alexandrium spp. isolates

**Figure 4:**
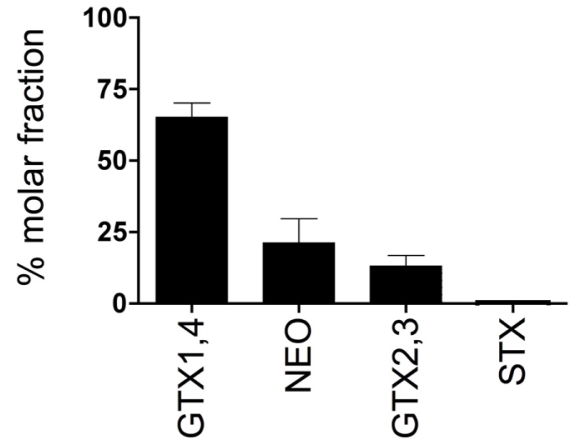
Mean (± sd) paralytic shellfish toxin (PST) profiles of four Sequim Bay *Alexandrium* spp. isolates quantified by high performance liquid chromatography (HPLC).

**Figure 5:**
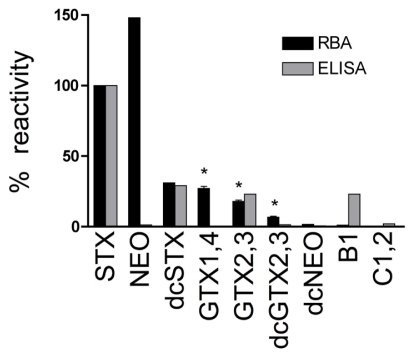
Percent reactivity of paralytic shellfish toxins (PSTs) quantified by receptor binding assay (RBA) and enzyme-linked immunosorbent assay (ELISA). Percent reactivity for saxitoxin (STX) is 100 % by definition (μg STX equiv./L) for both assays. * Indicate the standards that were analyzed at three concentrations in order to determine the standard deviation of the RBA (assay sd = 1.7 %). Reactivity data for ELISA is from Abraxis LLC, Warminster, PA.

The suite of PSTs identified in four *Alexandrium* spp. isolates from Sequim Bay consisted of GTX1,4, NEO, GTX2,3 and STX at 65 ± 10 %, 21 ± 17 %, 13 ± 7 % and 0.4 ± 0.3 %, respectively (Figure 4). The Abraxis ELISA microtiter plate kit recognizes STX and other PSTs at varying degrees. Cross-reactivities for the ELISA are < 0.2 % for GTX1,4, 1.3 % for NEO, 23 % for GTX2,3 and 100 % for STX (Figure 5). Due to the selectivity of the ELISA, the toxin profile can profoundly affect intracellular STX and extracellular STX levels quantified by the assay. Further analysis of a set of PST standards by receptor binding assay (RBA) in the present study revealed cross-reactivities of 100% (STX), 148 %(NEO), 31% (dcSTX), 27 ± 2.3% (GTX1,4), 18 ± 1.5% (GTX2,3), 6.7 ± 1.2% (dcGTX2,3), 1.7% (dcNEO), 1.2% (B1), and 0.4% (C1,2) (Figure 5). Saxitoxin has 100% cross-reactivity by definition because STX is the standard for both ELISA and RBA assays. A comparison of intra- and extracellular STX levels in four Sequim Bay isolates measured by both RBA and ELISA revealed that RBA values were consistently higher than values measured by ELISA in identical samples (Table 3). Specifically, RBA values were 33 ± 25 and 7.4 ± 1.8 times higher than ELISA values for intracellular and extracellular STX, respectively. This is likely explained by the fact that % cross-reactivities for GTX1,4 (the most abundant toxin in Sequim Bay isolates) were < 0.2 and 27 ± 2.3 for ELISA (Abraxis LLC, Warminster, PA) and RBA (this study), respectively (Figure 5).

## 4. Discussion and Conclusions

### 4.1 Fisheries and PSTs in Sequim Bay

The Sequim Bay region is ecologically important for marine fish and shellfish populations as well as economically and culturally important for local tribal communities that depend on this region’s natural resources for subsistence and livelihood. In addition to a long history of commercial, subsistence, and ceremonial harvesting Sequim Bay also has the longest documented history of PSTs in Washington State. Although PST levels in shellfish have been well documented, this is the first study to focus on both intra- and extracellular PST levels in relation to toxic cell densities. The fact that extracellular PSTs were detectable in field and culture samples in the present study suggests that extracellular PSTs are likely bioavailable to planktonic larvae and reveals the importance of characterizing the presence and interannual variability of extracellular toxins in order to fully assess exposure risks to marine fish and shellfish species.

### 4.2 Dissolved PST levels in field-collected seawater and culture media samples

In the present study, extracellular STX levels were detected in both field-collected seawater and culture medium in which *Alexandrium* spp. isolates were grown, thereby confirming that extracellular PSTs are present in the marine environment in association with toxic *Alexandrium* spp. blooms. Extracellular STX levels ranging from 12 to 31 μg STX equiv./L, as quantified by RBA, were measured in the culture media of nine Sequim Bay *Alexandrium* spp. isolates (Table 2). Previous cyanobacterial and dinoflagellate culture studies have reported slightly higher extracellular PST levels of ≈ 50 to 75 μg/L as measured by HPLC ^[9, 15]^. To our knowledge, extracellular PST values from field-collected seawater samples have not been reported. Our study reports a maximum extracellular STX level of 0.8 μg STX equiv./L in a Sequim Bay seawater sample as measured by ELISA (Figure 2). Our comparison of ELISA and RBA techniques revealed that the detection method profoundly affected the quantification of toxin levels. This is due to the selectivity of the assays as well as the composition of the toxin profiles in the samples. For example, GTX1,4, the most abundant toxin in the Sequim Bay isolates (Figure 4), had drastically different cross-reactivities of 27 ± 2.3 and < 0.2 % when quantified via RBA and ELISA, respectively (Figure 5). Not surprisingly, RBA consistently gave higher values than ELISA for both intra- and extracellular STX in our comparisons (Table 3). The ELISA is a structure-based assay, in that reactivity is dependent on recognition of antibodies generated to STX. The RBA is an activity-based assay in that reactivity is measured via displacement of tritiated STX from the pore of intact rat brain voltage-gated sodium channels and therefore likely gives a better estimate of potential toxicity. In previous studies, sodium channel radioreceptor assays have been positively correlated with mouse lethality bioassays in terms of predicting toxicity^[16]^. While this shows the limitation of the ELISA, its advantage is its sensitivity compared to both RBA and HPLC. For example, our field extracellular STX samples from 2006 were not quantifiable by RBA due to sensitivity issues, whereas they were quantifiable by ELISA. Clearly, when trying to characterize toxin levels in the marine environment, it is important to consider the best toxin detection method for the circumstances. The newly available PST ELISA kit, although more sensitive, underestimates toxicity of samples due to its substantially reduced cross-reactivity with common PSTs other than STX. However, it is possible that a correction factor for toxicity could be developed that takes into consideration the difference between structural and activity-based analyses. Undoubtedly STX quantifications via RBA are more relevant than quantifications made via ELISA techniques when trying to evaluate potential threats to exposed organisms.

### 4.3 Effects of extracellular PST exposure on fish larvae

Previous studies have confirmed that the dietary consumption of PSTs via algal or zooplankton vectors is an ecologically relevant route of PST exposure causing acute toxicity in adult and larval fish during toxic blooms ^[17–23]^. More recent laboratory studies have also demonstrated that extracellular STX exposure significantly impairs the physiology and behavior of developing fish larvae and may also be an ecologically relevant route of exposure. For example, extracellular STX exposures of approximately 200 μg STX equiv./L as measured via RBA caused a complete loss of sensorimotor function in zebrafish larvae ^[7]^. Exposures of up to 400 μg STX equiv./L were required for the same type of sensorimotor impairment in larval Pacific herring ^[8]^. In another study, extracellular STX levels as low as 10 μg STX equiv./L caused delayed hatching and levels of 500 μg STX equiv./L led to malformations and mortalities in zebrafish larvae ^[24]^.

Field extracellular STX levels measured in this study were not near those reported to impact larval development in previous studies, however, these values may have been underestimated due to their quantification by ELISA. Based on our data, ELISA underestimates toxin levels by as much as 61 times in intracellular STX samples and 8.5 times in extracellular STX samples. If we were to multiply our maximum field detected extracellular STX level of 0.8 μg STX equiv./L by 8.5, the resulting dSTX value of 6.8 μg STX equiv./L falls near the range of values that have been shown to delay hatching ^[24]^, but is still 40-fold lower than levels known to block sensorimotor function in larval fish in laboratory studies ^[7]^. Due to the patchiness of harmful algal blooms, it is likely that the concentrations of extracellular PSTs in the natural environment are highly variable, with elevated levels of toxin present in association with dense microlayers of phytoplankton or at the end of blooms when toxin-containing cells begin to die and lyse. Obviously, sampling only one site in Sequim Bay is not reflective of the highest levels of extracellular STX present in the marine environment. Further studies are needed to determine if extracellular STX exposure levels in the field reach those that can impact planktonic larvae. However, our data do confirm that extracellular PSTs are present in Sequim Bay, a known spawning site for planktivorous fish such as Pacific herring.

### 4.4 Frequency of PSP blooms and importance of monitoring in Sequim Bay

The levels of PSTs in shellfish and the number of associated shellfish closures have been increasing over the past five decades in Puget Sound, Washington ^[3]^. Sequim Bay in particular has the longest history of PSP blooms, which regularly occur during August and September (Figure 2). The presence of PSTs in shellfish at levels above the regulatory limit (80 μg STX equiv./100 g meat) in Puget Sound has been positively correlated with summer warming of sea surface temperature (SST) ^[25]^. Continued warming SSTs, potentially influenced by global climate change, will likely increase the temporal and spatial window for PSP blooms to occur ^[5]^. Due to the apparent increase in HAB frequency and intensity, monitoring for HAB toxins on a regular basis in Sequim Bay will continue to be important for managing natural resources and protecting human health.

Our comparison of PST extraction techniques in the present study provides useful information for monitoring entities in that it shows that extraction of intracellular toxin samples with dH_2_O results in similar toxin measurements compared to other traditional extraction protocols that have historically utilized acid (Table 1). This is convenient because it allows for the utilization of multiple analytical detection methods for multiple toxins and for comparisons between detection techniques, not all of which can tolerate acid. As HABs increase, the development of reliable and efficient toxin detection techniques as well as an understanding of how toxin quantification relates to actual toxicity become increasingly important.

### 4.5 Summary

In the present study, both extracellular and intracellular PSTs were present in field collected seawater samples and in cultured Sequim Bay *Alexandrium* spp. isolates, thereby confirming that marine planktonic larvae are likely exposed to both forms of PSTs during harmful algal blooms. Our data revealed that GTX1,4 was the dominant PST present in local *Alexandrium* spp. and that the toxin profile as well as the toxin detection techniques employed can profoundly effect the quantification of toxin levels. Further studies are needed to determine if extracellular PSTs are a result of active release from toxic cells and/or a consequence of cell lysis associated with bloom age or zooplankton feeding and if levels of extracellular toxins reach those that could negatively impact larval fish. In any case, the apparent increase in PSP bloom frequency and intensity in the Pacific Northwest, suggests that characterizing both extracellular and intracellular PST levels is important for accurately assessing HAB-related risks to marine life.

## Figures and Tables

**Table 1:** Intracellular saxitoxin (STX) concentrations (μg STX equiv. /L) with dH_2_O and 0.1 M acetic acid extraction methods using eight sets of duplicate samples from laboratory cultures (columns A & B). An aliquot from each extraction was further extracted with boiling HCl (columns C & D). Toxin levels were quantified by receptor binding assay (RBA). The coefficient of variation (CV) for samples analyzed by RBA was < 20%. Toxin values in samples extracted in dH2O were not significantly different from those in samples extracted in acid treatments (P = 0.05).

Sample	A. dH_2_O	B. 0.1 M acetic acid	C. dH_2_O/HCl	D. 0.1 M acetic acid/HCl
1	154	131	121	145
2	64	89	75	95
3	81	121	84	105
4	66	66	49	69
5	95	100	87	111
6	107	98	108	116
7	31	36	35	49
8	69	63	48	65

**Table 2:** Extracellular saxitoxin (STX) concentrations (μg STX equiv. /L) in filtrate from five separate *Alexandrium* spp. cultures filtered under normal (5 in. Hg) and light (2 in. Hg) vacuum pressures. Toxin levels were quantified by receptor binding assay (RBA) and cell densities are shown for convenience. The coefficient of variation (CV) for samples analyzed by RBA was < 20%. Toxin values were not significantly different between vacuum treatments (P = 0.05).

sample	Alexandrium cells/L	normal vacuum extracellular STX	light vacuum extracellular STX
1	3.5 x 10^6^	30	28
2	2.3 x 10^6^	24	27
3	2.2 x 10^6^	26	30
4	1.8 x 10^6^	23	31
5	2.9 x 10^6^	24	24

**Table 3:** Comparison of intra- and extracellular saxitoxin (STX) concentrations (μg STX equiv. /L) quantified in four Sequim Bay (SQ) *Alexandrium* spp. isolates measured by receptor binding assay (RBA) and enzyme-linked immunosorbent assay (ELISA). The Coefficients of variation (CV) were < 20 % and < 15 % for RBA and ELISA samples, respectively.

Sample	Cells/L	Intracellular STX (RBA)	Intracellular STX (ELISA)	Extracellular STX (RBA)	Extracellular STX (ELISA)
SQ-1	6.2 X 10^6^	61	1.0	14	2.9
SQ-2	6.8 X 10^5^	17	3.0	15	2.0
SQ-3	2.2 X 10^6^	127	6.8	29	3.4
SQ-4	3.3 X 10^6^	96	2.1	12	1.4
